# Biological variation and reference change values of serum Mac‐2–binding protein glycosylation isomer (M2BPGi)

**DOI:** 10.1002/jcla.24319

**Published:** 2022-03-13

**Authors:** Rihwa Choi, Gayoung Chun, Unyeong Go, Sang Gon Lee, Eun Hee Lee

**Affiliations:** ^1^ Department of Laboratory Medicine Green Cross Laboratories Yongin Republic of Korea; ^2^ Department of Laboratory Medicine and Genetics Samsung Medical Center Sungkyunkwan University School of Medicine Seoul Republic of Korea; ^3^ Department of Infectious Disease Green Cross Laboratories Yongin Republic of Korea; ^4^ Green Cross Laboratories Yongin Republic of Korea

**Keywords:** biological variation, health checkup, Korea, liver fibrosis, M2BPGi

## Abstract

**Background:**

Limited data are available with regard to biological variations of the Mac‐2–binding protein glycosylation isomer (M2BPGi), a liver fibrosis biomarker.

**Methods:**

Long‐term biological variation of M2BPGi was investigated using longitudinally measured M2BPGi test results from healthy Korean adult subjects. One‐way analysis of variance (ANOVA) tests were used to calculate the reference change value (RCV) of M2BPGi based on biological variation estimates. Furthermore, asymmetric RCV was calculated according to a recent publication of the European Federation of Clinical Chemistry and Laboratory Medicine Working Group on Biological Variation and Task Group for the Biological Variation Database (EFLM TG‐BVD).

**Results:**

A total of 363 test results from 174 Korean subjects undergoing general health checkups were requested from 13 local clinics and hospitals during a 38‐month period. The within‐subjects biological variation (CV_I_), between‐subject biological variation (CV_G_), analytical variation (CV_A_), RCV, and individuality index (II) values for serum M2BPGi were 23.3%, 30.0%, 4.3%, 65.6%, and 0.78, respectively. Asymmetric RCV calculated using formulae by a recent EFLM TG‐BVD publication ranged from −41.9 to 72.0%. Desirable analytical performance specifications for M2BPGi derived from biological variation were as follows: imprecision 11.6%, bias 9.6%, and total allowable error 28.7%.

**Conclusions:**

RCV based on biological estimates may be helpful for evaluating and interpreting serial M2BPGi measurements by physicians and in clinical laboratories.

## 1 INTRODUCTION

Biological variability data from an individual that changes in serial results may be due to an individual improving or deteriorating clinically, but may also be due to inherent sources of variation, including preanalytical variation, analytical imprecision (CV_A_), and within‐subject biological variation (CV_I_).^1^ Biological variability data is an important source of information to determine an allowable total error (TEa) limit in clinical laboratories.^1^ Reference change values (RCVs) represent a critical difference based on biological variation estimates and RCVs are one tool to investigate the significant differences in serial results from an individual that require additional review for test result verification as a quality improvement effort by laboratories.^2^, ^3^ Furthermore, in clinical practice for bone turnover markers as an example, RCVs based on biological variation are used as the term of least significant change for patient management in the treatment and monitoring of osteoporosis under clinical practice guidelines.^4^, ^5^

Meanwhile, Wisteria floribunda agglutinin‐positive Mac‐2–binding protein (WFA + M2BP; Mac‐2–binding protein glycosylation isomer, M2BPGi), has recently been introduced to clinical laboratories as a non‐invasive surrogate marker for liver fibrosis in chronic liver diseases such as viral hepatitis, nonalcoholic fatty liver disease, and hepatocellular carcinomas.^6^, ^7^ Serial measurements of M2BPGi can be used to estimate the progression and prognosis of liver fibrosis and predict hepatocellular carcinomas.^7^, ^8^ Studies focused on its variation with regard to clinical implications of M2BPGi are still ongoing.^7^, ^8^

Considering that the observed variation in M2BPGi results in an individual in a steady‐state is affected by the CV_I_ and CV_A_, not only by disease progression or regression, having knowledge on RCVs is helpful for clinical laboratories in terms of quality improvement as well as for physicians to understand the analytical aspects of the M2BPGi test and interpret results for patient management. Furthermore, analytical performance specifications based on biological variation data can improve the quality of clinical laboratory practices. However, information on RCVs for M2BPGi based on biological variation is limited.

Therefore, we investigated biological variation and RCV of M2BPGi in Korean patients. We also evaluated analytical performance specifications to improve quality in clinical laboratories using calculated biological variation estimates.

## 2 MATERIALS AND METHODS

### 2.1 Study populations

We retrospectively reviewed data obtained through the laboratory information system of Green Cross Laboratories between February 2018 and April 2021. Overall, 40,137 M2BPGi test results for health check‐ups performed in Korean adults (>19 years) visiting health promotion centers were obtained. Among them, results were excluded for (1) subjects with missing age or gender data, (2) subjects where M2BPGi was measured only once during the study period, and (3) subjects whose M2BPGi test results showed 1+ or 2+ at least once during a follow‐up in order to assess biological variation in subjects assumed to be relatively healthy. The M2BPGi test as a liver fibrosis biomarker in Green Cross Laboratories was installed and available for two test orders by physicians; one for diagnostic and monitoring purposes for patients with suspected liver fibrosis, and the other code is for relatively healthy subjects visiting health promotion centers throughout Korea. The results of this study were based on data from the latter. All data were anonymized prior to statistical analysis. This study was conducted according to the guidelines outlined in the Declaration of Helsinki, and all procedures involving human subjects were approved by the Institutional Review Board of Green Cross Laboratories (GCL‐2021–1025–01).

### 2.2 M2PBGi analysis

Serum M2BPGi was measured with an automated chemiluminescent enzyme immunoassay using an M2BPGi kit on a HISCL‐5000 analyzer (HISCL; Sysmex Co.) according to the manufacturer's instructions. M2BPGi tests results were presented as quantitative and qualitative values.^6^, ^8^ The measurement unit for the quantitative value was presented as a cutoff index (C.O.I.). Qualitative results were defined as 1+ positive when the C.O.I. was above the cutoff value of 1.00 ≤ C.O.I. <3.00, and 2+ positive when the C.O.I. ≥3.0 according to the manufacturer's instructions.^6^, ^8^

### 2.3 Statistical analysis

We used a one‐way analysis of variance (ANOVA) model for data analysis to investigate the CV_I_ and between‐subject biological variation (CV_G_).^9^, ^10^ CV_A_ was obtained from the internal quality control program of the laboratory (4.3%).^9^, ^10^ The individuality index (II) was calculated as follows^2^: II = CV_I_/CV_G_. The RCV for M2BPGi were calculated using the following equations: RCV = 2 ^1/2^ Z (CV_A_^2^ + CV_I_^2^) ^1/2^, where *Z* is the number of standard deviations appropriate to the desired probability.^2^ *Z*‐scores of 1.96 for *p* < 0.05 and 2.58 for *p* < 0.01 are often cited in works with regard to the generation and application of data on biological variation. In this study, *Z*‐scores of 1.96 for *p* < 0.05 were used at a 95% confidence interval. Statistical significance was defined as a value of *p* < 0.05. Tukey's method was used to detect outliers. Normal distribution among subjects was tested using the Shapiro‐Wilk test to assess skewness and kurtosis. Homogeneity of intra‐individual variances of M2BPGi was tested using Levene's test for equality of variances. Statistical analysis on data dependency on sex or age was tested using *t*‐test and Pearson correlation coefficient. In addition to one‐way ANOVA, asymmetric RCV was calculated according to following formulae from a recent publication of the European Federation of Clinical Chemistry and Laboratory Medicine Working Group on Biological Variation and Task Group for the Biological Variation Database (EFLM TG‐BVD): RCV = 100% (exp (± *Z* 2^1/2^(CV^2^_LnA_ + CV^2^_LnI_)^1/2^)‐1), where CV_Ln_ = (ln (1+CV^2^))^1/2^ and *Z* = 1.64 (probability level 95%).^11^

Analytical performance specifications for M2BPGi measurement derived from biological variation data were investigated using the following calculations. For optimal performance specifications: imprecision CV_A_ <0.25 CV_I_, bias <0.125 (CV_I_^2^ + CV_G_^2^)^1/2^, TEa <0.125 (CV_I_^2^ + CV_G_^2^)^1/2^ + 1.65 (0.25 CV_I_). For desirable performance specifications, the following calculations were used: imprecision CV_A_ <0.50 CV_I_, bias <0.250 (CV_I_^2^ + CV_G_^2^)^1/2^, TEa <0.250 (CV_I_^2^ + CV_G_^2^)^1/2^ + 1.65 (0.5 CV_I_). For minimal performance specifications: imprecision CV_A_ <0.75 CV_I_, bias <0.375 (CV_I_^2^ + CV_G_^2^)^1/2^, TEa <0.375 (CV_I_^2^ + CV_G_^2^)^1/2^ + 1.65 (0.75 CV_I_).^10^ Statistical analysis was executed using SAS version 9.4 (SAS Institue, Inc.).

## 3 RESULTS

During the 38‐month study period, 40,137 specimens were submitted to Green Cross Laboratories for M2BPGi analysis. Among them, 363 M2BPGi tests results from 174 Korean adults (130 men and 44 women) with a mean age of 47.3 (standard deviation [SD] 9.6) years were included after exclusion. Most subjects (92.0%) had two M2BPGi measurements during follow‐up. Each subject had two to four measurements of M2BPGi. The mean (SD) follow‐up period was 12.6 (7.5) months. General characteristics of the study population are summarized in Table 1. The M2BPGi C.O.I. values were different between men and women (*p* < 0.01). There was no data dependency by age based on Pearson's correlation coefficient in men (*p* = 0.02, *r* = 0.1423) and women (*p* = 0.14, *r* = 0.15). No outliers were detected based on Tukey's method. Normal distribution of M2BPGi among subjects was verified using the Shapiro–Wilk test with assessment of skewness (0.51) and kurtosis (−0.44). Levene's test for equality of variances for M2BPGi showed homogeneity (*p* = 0.39).

**TABLE 1:** General characteristics of the study subjects

	Total (*n* = 174)	Men (*n* = 130)	Women (*n* = 44)
Age, years (mean, SD)	47.3 (9.6)	46.6 (8.9)	49.5 (11.1)
Follow‐up numbers, *n* (mean, SD)	2.1 (0.3)	2.1 (0.3)	2.1 (0.3)
Follow‐up period, month (mean, SD)	12.6 (7.5)	13.7 (7.4)	9.4 (7.1)
M2BPGi, C.O.I. (mean, SD)	0.50 (0.19)	0.48 (0.18)	0.56 (0.2)

Abbreviations: C.O.I., cutoff index; SD, standard deviation.

**FIGURE 1:**
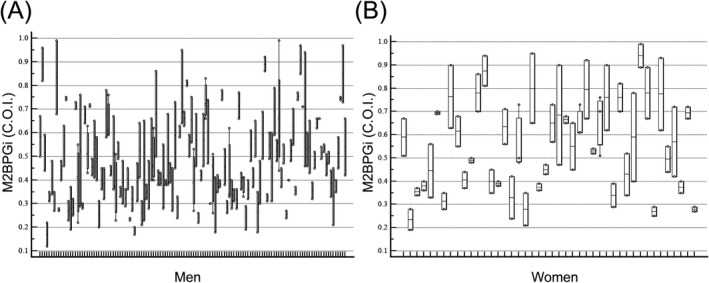
Individual changes in serial M2BPGi tests during follow‐up in (A) 130 men and (B) 44 women. The X‐axis represents individual cases by age (young to old; from left to right), and the Y‐axis represents quantitative the M2BPGi Cut‐off Index (C.O.I.). The bars represent the minimum and maximum values, the middle line represents the median, and the central box represents the values from the lower to upper quartile (25 to 75th percentiles)

Intraindividual changes of M2BPGi during a follow‐up to allow visual inspection are shown in Figure 1. Biological variation estimates of CV_A_, CV_I_, CV_G_, II, and RCV for M2BPGi using data for all subjects and analytical performance specifications for M2BPGi measurement derived from biological variation data are presented in Table 2. Asymmetric RCV calculated using formulae by a recent EFLM TG‐BVD publication ranged from −41.9% to 72.0%. Desirable analytical performance specifications for M2BPGi derived from biological variation were as follows: imprecision 11.6%, bias 9.6%, and TEa 28.7%.

**TABLE 2:** Variance components and analytical performance specifications based on biological variation estimates

Variance components		Analytical performance specifications			
CV_T_, %	23.7 (21.5–26.3)^†^	Quality level	Precision	Bias	TEa
CV_A_, %	4.3	Optimal performance, %	<5.8	<4.8	<14.4
CV_I_, %	23.3	Desirable performance, %	<11.6	<9.6	<28.7
CV_G_, %	30.0 (27.1 to 33.5)^†^	Minimal performance, %	<17.5	<14.3	<43.1
II	0.78				
RCV, %	65.6				
Asymmetric RCV, %^‡^	−41.9–72.0				

Note

Variance components calculated from the 363 M2BPGi test results from 174 total subjects. ^†^95% confidence interval. The 95% confidence interval of CV_A_ could not be determined because CV_A_ was calculated using data from an internal quality control program not using replicated test results of clinical specimens. Therefore, the 95% confidence interval of CV_I_ derived from the subtraction of CV_A_ from CV_T_ could not be calculated. ^‡^Asymmetric RCV was calculated according to a recent publication of the European Federation of Clinical Chemistry and Laboratory Medicine Working Group on Biological Variation and Task Group for the Biological Variation Database. CV_A_, analytical coefficient of variation; CV_I_, within subject biological variation; CV_G_, between‐subject biological variation; CV_T_, total variation; II, individuality index; RCV, reference change value; TEa, total allowable error.

## 4 DISCUSSION

In this study, we investigated the long‐term biological variation and RCV of M2BPGi (a liver fibrosis biomarker) using data from Korean adults who visited local clinics and hospitals for health check‐ups in a practical way in clinical laboratories. Furthermore, analytical performance specifications for quality goals based on biological variation estimates were also investigated.

M2BPGi can be measured serially during the assessment of liver fibrosis to monitor liver function, the effects of antiviral therapy, liver failure, and the development of cancer.^7^ Serial M2BPGi test results can be interpreted based on knowledge of the natural history of liver fibrosis progression as well as inherent sources of variation including preanalytical factors, CV_A_, and CV_I_^1^ RCVs derived from biological variation, can be used to calculate limits for the maximum amount of random variation in results that would be expected in an individual over a period of time.^2^ According to Clinical And Laboratory Standards Institute (CLSI) guidelines, three approaches have been used to set delta check limits: (1) empirical, (2) from the distribution of delta values in the laboratory patient population (ideal approach), and (3) simulations of misidentified specimens.^2^ Considering that information with regard to M2BPGi RCV in the literature is limited, results from the present study may be useful for laboratories to set empirical delta check limits. Furthermore, this study can provide a practical way to investigate and compare limits of delta checks using their own data for clinical laboratories. Meanwhile, M2BPGi presented as a qualitative result based on a quantitative C.O.I. value, the qualitative cutoff concentrations may also be used as a delta check limit. The percentage change was large despite small changes in C.O.I. values (RCV 65.6%) because the quantitative values of M2BPGi in our data was low (<1.0 C.O.I. for all measurements).

Biological variation can be estimated using statistical models of direct methods using prospective studies and indirect methods using retrospective studies with different strengths and limitations.^12^ The Working group on Biological Variation of the EFLM generated a prospective study specifically designed to calculate biological variation estimates, the multicenter European Biological Variation Study (EuBIVAS).^13^, ^14^ However, most clinical laboratories have limited resources to design and perform prospective studies to attain quality management using BV estimates.^1^, ^2^ The strength of this study is the novelty of M2BPGi biological variation estimates in healthy subjects. Biological variation can be used to obtain biological estimates of M2BPGi in a practical way via retrospective review of longitudinally measured data from subjects visiting local clinics and hospitals for health checkups. However, the retrospective design of this study could also be a limitation. Use of indirect methods for estimating biological variation using retrospective data had limitations in decreasing the robustness of results including non‐standardized preanalytical variables; the homeostatic status of the subjects is unknown; CV_A_ is derived from data of quality control materials (with a different matrix from clinical specimens of patients) instead of repeated measurement of clinical specimens of study subjects; limited number of samples per subject; and variable sampling intervals.^12^ The statistical methods used for analysis of biological estimates using retrospective big data such as methods by Bhattacharya et al. could not be applied because of limited numbers of study subjects.^15^ The results of our study may not be generalizable to other ethnic populations because all subjects within this study were Korean adults. However, this study includes the use of an asymmetric RCV approach proposed by a recent EFLM TG‐BVD publication that identifies different RCVs for rising and falling values of a measurand.^11^ The BV estimates of M2BPGi assessed with different statistical models and methods using prospective and retrospective studies could be compared through future studies performed in different ethnic populations.^16^ Considering that some biological variation may depend on health status and may change in a short time period, future studies with a prospective design and detailed clinical information in association with liver fibrosis are needed to understand more accurate implications of changes in M2BPGi.^3^

In conclusion, we evaluated long‐term biological variation in M2BPGi (a liver fibrosis biomarker) using data from Korean adult subjects visiting local clinics for health checkups. The results of this study may be helpful to both physicians using M2BPGi tests for patient management and clinical laboratories. Biological estimates of M2BPGi and analytical performance specifications based on their assessment in the present study may be a good source to define error limits for other clinical laboratories to implement and manage quality systems.

## CONFLICT OF INTEREST

All the authors declare that there is no conflict of interests.

## AUTHOR CONTRIBUTIONS

This work was performed as a collaboration among all the authors. All the authors accept responsibility for the entire content of this submitted manuscript and approve its submission. All authors contributed to manuscript preparation; R. Choi, G. Chun, U. Go, S. G. Lee and E. H. Lee collected the data or contributed to data analysis; R. Choi, and G. Chun designed the study; R. Choi, S.G. Lee and E. H. Lee had full access to all the data in the study and takes responsibility for the integrity of the data and the accuracy of the data analysis. All authors read and approved the final manuscript.

## Data Availability

The data that support the findings of this study are available from the corresponding authors upon reasonable request.
